# Continuous glucose monitors and virtual care in high-risk, racial and ethnic minority populations: Toward promoting health equity

**DOI:** 10.3389/fendo.2023.1083145

**Published:** 2023-01-25

**Authors:** Elizabeth A. Vrany, Felicia Hill-Briggs, Patti L. Ephraim, Alyson K. Myers, Patricia Garnica, Stephanie L. Fitzpatrick

**Affiliations:** ^1^ Institute of Health System Science, Feinstein Institutes for Medical Research, Northwell Health, Manhasset, NY, United States; ^2^ Fleischer Institute for Diabetes and Metabolism, Montefiore Medical Center, Albert Einstein College of Medicine, Bronx, NY, United States; ^3^ Donald and Barbara Zucker School of Medicine at Hofstra/Northwell, Hempstead, NY, United States; ^4^ Department of Medicine, North Shore University Hospital, Manhasset, NY, United States

**Keywords:** diabetes, continuous glucose monitor (CGM), disparities, virtual care, race & ethnicity

## Abstract

Continuous glucose monitors (CGMs) have become an important tool to aid self-management of blood glucose for many patients with diabetes in the U.S., and the benefits of CGM use are well-documented. However, disparities in CGM use exist, with lower use in certain marginalized racial and ethnic groups. CGM may be an important and underutilized tool to help reduce inequities. Evidence supporting the use of CGMs as a part of virtual care is discussed, with an emphasis on designing virtual diabetes care programs to promote health equity. Recommendations for clinical practice and research are presented. In clinical practice, CGM should be an option for all people with diabetes who qualify based on clinical practice guidelines, regardless of race, ethnicity, or other individual characteristics. Future research should characterize the use of, benefit from, and preferences for CGM among individuals from racial and ethnic groups to guide interventions at the health system, clinic, provider, and patient levels to promote equitable, evidence-based, and guideline-directed CGM use in marginalized racial and ethnic groups with diabetes.

## 1 Introduction

Approximately 37 million people in the U.S. had diabetes in the year 2021 (1). Decades of research have documented health disparities in diabetes, with individuals from marginalized racial and ethnic groups experiencing excess risk of diabetes incidence, prevalence, complications, and mortality (2). Improving diabetes management and outcomes in populations of health inequity is a priority for research and public health organizations (3–5). Recent studies demonstrate that, while rates of diabetes-related complications are decreasing in the U.S., rates continue to rise in Black and Hispanic persons with diabetes (6, 7). Lowering blood glucose is directly associated with lower rates of diabetes complications (8), making self-monitoring of blood glucose a key component of diabetes management (9). Continuous glucose monitoring (CGM) has emerged as an important tool to support self-monitoring of blood glucose and may be an important tool to help reduce inequities.

Well-conducted, large randomized controlled trials and prospective studies demonstrate that CGM improves A1C, reduces diabetes-related hospitalizations and emergency room visits, reduces the frequency of dysglycemia, reduces diabetes distress, and improves quality of life in people with diabetes on intensive insulin regimens (10–16). In addition to improving health and well-being, CGMs offer a simplified, automated approach to blood glucose monitoring that removes many hassles of daily diabetes self-management.

Recent reviews have summarized that CGM use is lower in Black/African American and Latinx American populations, relative to the White American population (17, 18). These same marginalized groups engage in lower rates of self-monitoring of blood glucose (19) and face challenges in traditional health care due to limited access to and quality of care, racism and bias in care, and social determinants. CGMs may be an important and underutilized tool to help reduce inequities.

In this paper, we summarize disparities in CGM use, barriers to equitable CGM use, and opportunities for using CGM in diverse populations as a part of virtual diabetes care to help reduce inequities. Additionally, we identify knowledge gaps and provide recommendations for research and clinical practice to promote equitable and guideline-directed diabetes care that leverages CGM, particularly as a part of virtual care.

## 2 Clinical practice guidelines and indications for CGM use

Several clinical practice guidelines developed by diabetes-focused professional organizations provide recommendations for CGM use for people with diabetes (20–22). The American Diabetes Association’s (ADA) Standards of Medical Care in Diabetes recommends that CGM should be offered to adults and youth with diabetes on multiple daily injections or continuous subcutaneous insulin; they additionally recommend that CGM can be used by adults with diabetes on basal insulin (20). In consensus, the American Association of Clinical Endocrinology’s (AACE) Clinical Practice Guidelines state that CGM is recommended for all persons with diabetes treated with intensive insulin therapy, and CGM may be recommended for individuals with type 2 diabetes (T2D) who are treated with less intensive insulin therapy (21). Uniquely, AACE’s guidelines recommend CGM for individuals with problematic hypoglycemia. Although some recommendations vary across guidelines, CGM is consistently recommended for individuals with diabetes who are treated with intensive insulin regimens, with stipulation that treatment using CGM should be individualized and be offered to those who are willing and capable.

Practice guidelines are clear that CGM use is beneficial for people with T1D across the lifespan (23), and CGM adoption is increasingly common for many people with T1D (24). Practice guidelines are not definitive regarding CGM use in people with T2D. A few clinical trials in people with T2D on intensive insulin regimens have demonstrated that CGM improves hemoglobin A1C and reduces hypoglycemia (12, 14), but little is known about benefits of CGM in individuals on noninsulin or less intensive insulin regimens (25). However, The ADA’s Standards of Care state that routine glucose monitoring may be helpful for adults with T2D who are not on insulin to elucidate the impact of diet, activity, and medication on glucose levels (20).

## 3 Disparities in CGM use

Despite clinical practice guidelines endorsing CGM use and strong evidence demonstrating the benefit of CGM, rates of CGM adoption remain low, particularly in marginalized groups. Recent reviews summarizing disparities in diabetes technology use conclude that rates of CGM use vary by race and ethnicity, with lower use in historically marginalized racial and ethnic populations (17, 18). To expand upon these reviews, characteristics of the extant studies examining CGM use by race and ethnicity are reported in **Table 1**. Only one new study has been published since the most recent review in this area (2022) (17). Kanbaour et al., 2023 conducted a retrospective clinic-based cohort study of 1,258 adults with T1D who received care between 2013-2020 (28). The authors report that, relative to non-Black adults, Black adults were less likely to use CGM at baseline and were less likely to initiate CGM over the study period. This study aligns with prior studies in this area, which demonstrate that CGM use is lower in non-Hispanic Black and Hispanic individuals with T1D across all age ranges, relative to non-Hispanic Whites (26, 27, 29, 30). As explained by Agarwal et al., 2022, these disparities persist after adjusting for socioeconomic status, education level, insurance, health literacy, numeracy, diabetes clinical outcomes and management factors, and care setting (17). Therefore, lower use in people with T1D from these marginalized racial and ethnic groups occurs independently of objective clinical decision-making factors.

**Table 1:** Characteristics of studies examining CGM use by race and ethnicity.

Authors	Study Design	Study Participants & Setting	Analysis	Adjustment for Covariates	Results
Agarwal et al., 2021 (26)	Cross-sectional	300 young adults (18-28 years) with T1D recruited from six T1D Exchange clinic sites (the Young Adult Racial Disparities in T1D Study)	% CGM use by race and ethnicity; statistical differences between groups determined by χ^2^ test; % CGM use by race and ethnicity with adjustment based on multivariate logistic regression	Demographic factors, socioeconomic status, insurance status, health literacy, clinic attendance, care site, and diabetes-management factors	CGM use was lower among Black (28%) and Hispanic (37%) than among White (71%) young adults (*p*s <.001); there were no differences between Black and Hispanic young adults. After adjustment for covariates, percentage differences between groups attenuated; CGM use was lower among Black (31%) than among White (53%) and Hispanic (58%) young adults^±^.
Foster et al., 2019 (27)	Retrospective study	22,697 adults and children (1-93 years) enrolled in the T1D Exchange clinic registry from 2016-2018	% CGM use stratified by race and ethnicity, age category, and income level	No adjustment Results are stratified by age and income	CGM use was lower among Black than among White adults across all age ranges and income levels.
Kanbour et al., 2023 (28)	Retrospective clinic-based cohort	1,258 adults (≥18 years) with T1D who received care at a comprehensive diabetes center clinic from 2013-2020	% CGM use by race and ethnicity; statistical differences between groups determined by χ^2^ test; Multivariate logistic regression with race (Black vs. non-Black) and covariates as IVs and CGM discussions and prescribing by a physician as DVs in separate analyses	Demographic factors, employment status, neighborhood status, insurance type, number of diabetes visits, other diabetes technology use, tobacco use, substance use, anxiety/depression, diabetes-related clinical values	Black adults were less likely than non-Black adults to use CGM at baseline (7.9% vs. 30.3%), initiate CGM over the study period (43.6% vs. 72.1%), discuss CGMs with their provider (79.6% vs. 91.7%), and be prescribed a CGM (50.0% vs. 68.4%; all *p*s <.001). In multivariate logistic regression analysis, Black adults were less likely to discuss CGMs with their provider (OR = 0.51; 95% CI 0.29, 0.90) and be prescribed a CGM (OR = 0.61; 95% CI 0.41, 0.93) than non-Black adults^±^.
Lai et al., 2021 (29)	Retrospective chart review	1,509 children (<17 years) with T1D who received care at an urban children’s hospital from 2015-2018	Multivariate logistic regression with race and ethnicity and covariates as IVs and CGM initiation as DV	Insurance type, age of diagnosis, and sex	CGM initiation was more frequent among White than among Black (OR=2.2, 95% CI=1.6-3) or Hispanic children (OR = 2.0, 95% CI 1.3-3) ^±^.
Fantasia et al., 2021 (30)	Retrospective chart review	227 adults (≥18 years) with T1D seen in an Endocrinology clinic in a safety-net hospital from 2016-2017	% technology use by race and ethnicity; Multivariate logistic regression with race and ethnicity and covariates as the IVs and CGM use as the DV	Age, language, insurance, and annual income	Technology use was lower in Black adults (OR = 0.25, 95% CI = 0.11-0.56) and “Other” race or ethnicity adults (OR = 0.30, 95% CI = 0.11-0.78) than among White adults^±^.

CGM, continuous glucose monitor; DV, dependent variable; IV, independent variable; T1D, type 1 diabetes; ^±^, adjusted analysis.

### 3.1 Barriers to equitable CGM use

Factors that have the potential to cause disparities in CGM use among people with T1D and T2D have been proposed (17, 18), including provider, health system/structural, and insurance barriers that cause people with diabetes from marginalized racial and ethnic groups to have less access to CGMs.

Healthcare providers hold an important responsibility to educate patients about their treatment options and engage with patients in shared decision-making. Bias, both implicit and explicit, may contribute to providers’ perceptions of patients’ interest, willingness, capacity, and financial ability to obtain and effectively use CGM devices. Provider implicit bias has been documented across a variety of provider and patient populations (31). Of relevance to CGM use, a few studies document provider implicit bias to recommend diabetes technology based on insurance (32, 33) and race or ethnicity (33). Relatedly, a recent clinic-based retrospective study demonstrated that, relative to non-Black adults, Black adults with T1D were less likely to discuss CGMs with their providers and be prescribed a CGM than non-Black adults (see **Table 1**) (28). It is plausible that providers may eliminate CGM as an option for members of marginalized groups based on biases, stereotypes, and generalizations regarding factors such as health literacy, socioeconomic status, and social contexts affecting their ability to take on new treatment regimens; however, this is an area requiring further study. Critically, these perceived barriers to using CGM are the same reasons why CGM is important to use in marginalized populations with diabetes who may benefit from automated and simplified daily diabetes routines.

People with diabetes may not be aware that CGM is an option or that insurance may cover the cost of the device. This may be especially the case among marginalized populations with limited healthcare access and suboptimal quality of care (34–38). Social determinants of health are systemic, structural barriers caused by the conditions in which people are born, grow, work, live, and age (39). Social determinants of health include socioeconomic status, neighborhood and physical environment, food environment, health care access/affordability/quality, and social contexts (40). In the U.S., these social determinants adversely affect marginalized populations and are directly associated with worse diabetes-related outcomes (40). In the setting of structural barriers to optimal diabetes management, it is even more imperative that the most effective treatment tools, including CGM, be made available.

The high cost of CGM and restrictive insurance policies are a barrier to CGM use. Based on data from the T1D Exchange, the most common barriers to CGM initiation and use are the cost of CGM and insurance coverage (41, 42). Insurance policies impose restrictions on who is eligible for CGM and require rigorous documentation from providers to demonstrate medical necessity (43, 44), requiring patients to have high-quality and consistent care by knowledgeable providers to facilitate CGM insurance coverage. Additionally, some insurance policies require patients to obtain CGMs through durable medical equipment suppliers (43), rather than through pharmacies in local communities. There is evidence demonstrating that obtaining CGM as a pharmacy benefit is faster than through durable medical equipment companies, thus reducing time-to-initiation of CGM (45). As added challenges, insurance policies for CGM coverage vary by insurance provider and evolve in response to advances in diabetes technology and most recently the COVID-19 pandemic. In response to the pandemic, the Centers for Medicare and Medicaid (CMS) updated policies to reduce barriers to CGM access by eliminating requirements for in-person visits, lab tests, and documented finger sticks (46). However, it is unclear whether these changes will persist, and challenges remain (47). Some private insurance does not cover CGM for T2D (48). Emerging evidence indicates that access to CGM varies by region within the US due, in part, to insurance coverage (49). Illustratively, Southeast states (e.g., Texas, Arkansas, Mississippi) have the lowest CGM use through Medicaid in the US (49). Variable and limited use of CGM in Medicaid beneficiaries may be due to variability in policies by state (43). As of 2022, Medicaid in 40 states covers CGM in some capacity, with variability in coverage based on diabetes-specific documentation (e.g., documentation of hypoglycemic episodes, hypoglycemia unawareness, and insulin pump use), prescriber qualifications (e.g., some states limit to endocrinologists only), need for preauthorization, coverage for people with type 2 diabetes, coverage for children, and locale of prescription fill (durable medical equipment supplier versus pharmacy). In July 2021, the requirement of documenting 4 blood glucose measurements *via* fingerstick per day was eliminated to increase access to CGM, particularly in the context of the COVID-19 pandemic. Medicaid policies by state are discussed comprehensively in a report from the Center for Healthcare Strategies (43). Critically, Medicaid enrollees are least likely to use a CGM, with particularly low rates of use among Black Americans and Hispanic individuals (50), highlighting the potential impact of insurance policies on CGM use disparities.

## 4 CGM use in virtual diabetes care

The COVID-19 pandemic precipitated an abrupt shift toward virtual care for ambulatory health services. In the post-pandemic era, there continues to be a role for telehealth and health technology, which improve care in some instances and circumvent barriers such as limited access, transportation, or time to attend medical visits (51, 52). Clinical practice guidelines for diabetes recommend visits with a provider every 3-6 months to measure hemoglobin A1C, conduct a physical exam, measure vitals, and review the treatment plan (53). It has been proposed that telehealth can reduce the frequency of in-person visits for some patients with diabetes (54). However, telehealth limits the physician’s ability to conduct physical exams and measure clinical values. To augment telehealth, there has been interest in the use of technology for remote patient monitoring.

In diabetes virtual care, CGM devices allow for remote monitoring of blood glucose. Blood glucose values can automatically be collected, uploaded, and accessible to providers, allowing for real-time monitoring between visits and providing a wealth of data to guide treatment decision-making. Moreover, time spent interpreting CGM data is billable through insurance, promoting the sustainability of provider review of blood glucose records (44). For people with diabetes, CGM as a part of diabetes virtual care has the potential to empower patients to leverage their blood glucose data to guide daily decisions about diabetes self-management behaviors between visits.

Evidence suggests that it is feasible and acceptable to implement CGM remotely *via* telehealth without the need for in-office visits. A qualitative study among parents of youth with T1D demonstrated that telehealth CGM initiation was well-accepted (55). Another study in a small sample (n=34) of predominantly White (85%) adults with T1D and T2D using insulin demonstrated that the telehealth CGM initiation, delivered by a diabetes educator, was feasible and improved A1C and diabetes distress (56). Additionally, a study among adults with T2D found that a virtual diabetes clinic that incorporated a mobile application, telehealth visits with an endocrinologist, and CGM use improved A1C and reduced hyperglycemia and diabetes distress (57, 58). These findings suggest that virtual models of diabetes care leveraging CGM can work, although larger trials should be conducted in more representative samples. It is the case, however, that in practice CGM initiation is frequently done *via* self-initiation with online video instruction and education provided by the device manufacturers.

## 5 Disparities in smartphone ownership and internet access: Potential impact on CGM use

Although CGMs can operate without a smartphone or internet access (i.e., by using a reader to obtain glucose data from the CGM sensor), CGM use is optimal when people can view their glucose data on their smartphones and share their blood glucose data with their providers using an internet connection. Therefore, the use of continuous glucose monitors relies, in great part, on access to and proficiency with using smartphones and the internet. Rates of smartphone ownership and internet access in the US are increasing, but unique trends that vary by race and ethnicity and location warrant attention (see **Table 2**). Within the last decade, rates of smartphone ownership increased from 56% in 2013 (59) to 81% in 2019 (60). In 2019, rates of smartphone use were generally similar across race and ethnicity groups, but use appeared lower among rural relative to urban and suburban locations. In contrast, broadband internet access has remained relatively stable over time, with only slight increases between 2013 (61) and 2019 (60). Notably, internet access at home appears lower in Black or African American and Hispanic individuals relative to White individuals and lower in rural relative to urban and suburban locations. There has been a stark increase over time in “Smartphone Only” internet use. Between 2013 (61) and 2019 (60), rates of accessing the internet at home with only a smartphone increased from 8% to 17% among US adults, with higher rates in Black or African American and Hispanic individuals relative to White individuals and higher rates in rural and urban relative to suburban locations. In sum, rates of smartphone use are on the rise. Although members of racial and ethnic minoritized groups continue to have limited broadband internet access, they are emergingly accessing smartphones and relying on their smartphones for internet access from home.

**Table 2:** Smartphone ownership and internet access patterns in the U.S. by race and ethnicity and by location, 2013-2019.

	Smartphone Ownership^†^		Broadband Internet Access at Home^†^		“Smartphone Only” Internet Use^†^		
	2013	2019	2013	2019	2013	2019	
US Adults, %	56	81	70	73	8	17	
Race/Ethnicity							
White, %	53	82	74	79	8	12	
Black or African American, %	64	80	62	66	10	23	
Hispanic, %	60	79	53	61	16	25	
Location							
Urban, %	59	83	70	75	9	17	
Suburban, %	59	83	73	79	7	13	
Rural, %	40	71	62	63	9	20	

^†^Data obtained from the Pew Research Center (60-62).

Trends in smartphone ownership and internet access should be considered as efforts are taken to promote equitable CGM use and diabetes technology use. Health care team members should discuss smartphone and internet access with patients when collaboratively evaluating the option of using CGM. Researchers using CGM in their studies should confirm smartphone ownership and internet access for their participants and, in cases where access is limited, provide connected devices to circumvent selective recruitment based on access. Health systems and policymakers should attend to these trends and disparities in the use of and access to devices and the internet, particularly as technology and telehealth continue to become an important part of healthcare delivery.

## 6 Equitable virtual care in diabetes

It is a common assumption that virtual care models have the potential to address barriers faced by marginalized populations. For instance, virtual care has the capacity to improve access to health care providers and clinics, eliminate transportation barriers, and allow appointments to be conducted where people live and work, thus reducing conflicts due to work schedules and personal/family responsibilities. Yet, it has been documented that virtual care can increase healthcare disparities (62–64). Commonly discussed is the “digital divide,” a term that describes disparities in access to digital devices and internet connection (65). Even among those with access to devices, there are further disparities in digital literacy (i.e., knowledge and skills to use technology effectively) (66–68) that may contribute to disparities in technology use outcomes. Moreover, accessing and using CGM technology may be limited by language barriers and device compatibility, as some CGM applications are available in English only and are compatible with a limited range of smartphone devices and operating systems (69).

To prevent disparities in access to, use of, and outcomes of virtual care, telehealth and health technology should be intentionally designed to promote equity. Weiss et al. report that the impact of health technology on health disparities depends on a particular community’s context and pathways through which they use and access the technology (70). Additionally, African American individuals expressed that past abuses by the U.S. medical system affect their views on new and innovative medical care (71). Shaw et al. provide recommendations to improve health equity in virtual care in the context of COVID-19 (64). Key recommendations were to engage marginalized community members in the planning and evaluation of virtual care programs, simplify complex interfaces and workflows, and leverage supportive intermediaries to help patients engage with virtual care. These recommendations are applicable to integrating CGM use in a virtual care environment with marginalized groups.

## 7 Recommendations for research and clinical practice

In order to design virtual care models using CGM that are effective and meaningful for people with diabetes from marginalized racial and ethnic groups, we must first characterize rates of CGM use, benefits of CGM use, and patient preferences around CGMs and diabetes virtual care, within each race and ethnic group. Research funding should be directed specifically to supporting research in marginalized populations. Consistent with these needs, research recommendations are summarized in **Table 3** and described below.

**Table 3:** Research and clinical recommendations for CGM use in marginalized populations as a component of diabetes virtual care.

Domain	Specific Recommendations
Research Recommendations	1. Characterize the rates of CGM use within marginalized groups with T1D and T2D, including Black or African American, Native American, Latinx American, and Asian American groups. 2. Characterize the effect of CGM use on diabetes-related clinical, behavioral, and psychosocial outcomes within marginalized groups, including Black or African American, Native American, Latinx American, and Asian American groups. 3. Conduct qualitative research to understand diverse patient perspectives of CGM use and diabetes virtual care. 4. Design, evaluate, and implement culturally relevant and meaningful interventions for CGM use as a component of virtual care, based on the formative research, above, and in alignment with clinical practice guidelines
Clinical Recommendations	1. Develop population-based approaches to: a) systematically provide education about the option of CGM to all people with diabetes to support shared decision-making related to imitating CGM, and b) systematically identify patients who may qualify for CGM based on clinical practice guidelines, regardless of race, ethnicity, or other individual characteristic. 2. Deliver evidence-based and meaningful education and support programs for CGM initiation and maintenance that are tailored to the needs, preferences, and challenges of that individual and their community. 3. Design diabetes virtual care models to promote equity by involving marginalized community members in the planning and evaluation of virtual care to ensure the programs align with the community’s needs and preferences. 4. Incorporate CGM into diabetes virtual care to augment remote monitoring of blood glucose for providers and patients to leverage as a part of shared-decision-making, treatment planning, and daily diabetes management.

CGM, continuous glucose monitoring, T1D, type 1 diabetes, T2D, type 2 diabetes.

First, research is needed to examine the rates of CGM use within marginalized groups with T1D and T2D, including Black/African American, Native American, Latinx American, and Asian American groups. Although studies of disparities in CGM use provide a signal of low use in some groups with T1D, no study has reported rates of use in Native American and Asian American groups with T1D, and no study has reported rates of use in people with T2D by race and ethnicity.

Second, research is needed to characterize the effect of CGM use on diabetes-related clinical, behavioral, and psychosocial outcomes within marginalized populations, including Black or African American, Native American, Latinx American, and Asian American groups. It is well-established that CGM improves clinical and behavioral outcomes on the aggregate, but, to our knowledge, no study has reported the benefits of CGM in each racial and ethnic group. This represents a critical gap in our understanding of the potential benefit of CGM in diverse communities with diabetes.

Third, there is a need to conduct qualitative research to understand diverse patient perspectives on CGM use and diabetes virtual care. Soliciting patient perspectives will elucidate the preferences, barriers, and needs of diverse communities related to the use of diabetes technology and telehealth, which will guide the development of interventions and clinical operations at the health system, provider, and patient levels to promote equitable, guideline-directed care using CGMs. Illustratively, in a qualitative analysis of Black and Latinx individuals who dropped out of a diabetes telehealth study, themes emerged around disinterest, inconvenience, and lack of perceived benefit (72). In the broader diabetes literature, qualitative studies document patient preferences and perspectives. A study among African American adults with diabetes identified that shared decision-making was affected by providers’ bias, discrimination, and cultural discordance as well as patients’ mistrust of White physicians and internalized racism (73). A study among predominantly Mexican American people with diabetes reported that the telephone-based intervention approach may be impersonal and may impede the establishment of a trusting bond (74). Additionally, providers’ cultural and linguistic competence is essential to develop a trusting patient-provider relationship for Hispanic adults with diabetes (75). Another qualitative study reported that African American and Latino individuals share concerns about confidentiality and the physical absence of the provider in telemedicine (71). This collection of findings provides insights, but future qualitative research should directly examine preferences related to CGM use and diabetes virtual care.

Finally, preliminary evidence demonstrates that CGM can be initiated *via* telehealth (56) and that diabetes virtual care that incorporates CGM is feasible and improves outcomes (57, 58). However, there is a need to design, evaluate, and implement culturally relevant and meaningful interventions for CGM use as a component of virtual care, based on the formative research, above, and in alignment with clinical practice guidelines.

In clinical practice, increasing CGM access and use in diverse populations will require widespread changes for health systems, clinics, and providers. Fundamentally, CGM should be offered to all patients who may qualify based on clinical practice guidelines, regardless of race, ethnicity, or other individual characteristics. Implicit bias and discrimination in health care may impact providers’ prescribing practices for diabetes technology (32, 33), even among qualified and well-meaning providers. Interventions to reduce bias in care increase provider awareness but do not result in sustained behavior change (76). To circumvent provider bias in CGM prescription, population-based approaches can be developed to systematically provide education about the option of CGM to all people with diabetes and identify the population of patients who may qualify for CGM based on clinical practice guidelines. For instance, patient registries can be developed from the electronic medical record to identify patient populations (e.g., diagnosed with T1D or T2D and on intensive insulin regimens). Members of the health care team can engage with every patient with diabetes to provide education on the option of CGM and its benefits/limitations to empower patients with knowledge to effectively engage with providers in shared decision-making.

For patients who will initiate CGM, the healthcare team should deliver evidence-based, meaningful education and support programs for CGM initiation and maintenance that are tailored to the needs, preferences, and challenges of that individual. Members of the healthcare team who engage patients in these conversations should be culturally aware and knowledgeable about CGM. Social determinants of health should be assessed and incorporated into interventions, as they influence many facets of diabetes treatment and decision-making.

Marginalized populations face barriers to obtaining high-quality care. The shift to virtual care in the wake of the COVID-19 pandemic presented an opportunity to address these barriers through telehealth and technology. Diabetes virtual care should be designed to promote equity by involving marginalized community members in planning and evaluation to ensure the programs align with the community’s needs and preferences. Virtual care should consider device access and digital literacy and should engender a trusting relationship in the absence of in-person interaction. CGM devices can be incorporated into diabetes virtual care to augment remote monitoring of blood glucose for providers and patients to leverage as a part of shared decision-making and diabetes management.

## 8 Conclusions

Disparities in access to and use of CGM in historically marginalized racial and ethnic populations contribute to widening of, rather than reduction in, long-standing disparities in diabetes outcomes in the U.S. It is well-established that CGM use improves the health and well-being of many patients with diabetes (11, 15, 16). However, there is a need to increase access to CGM and to characterize the use and potential benefit of CGM use in diverse populations.

The causes of disparities in CGM use are complex and multifactorial, and strategies to address these disparities will require widespread changes, including policy changes, with multilevel interventions at the health system, provider, and patient levels. Yet, CGMs may be particularly beneficial for marginalized populations with diabetes, who stand to benefit the most from improved blood sugar management and simplified, automated approaches to daily diabetes management. CGMs may be an important and underutilized tool to help reduce inequities in diabetes care and outcomes, particularly when used in virtual diabetes care.

## Data availability statement

The original contributions presented in the study are included in the article/supplementary material. Further inquiries can be directed to the corresponding author.

## Author contributions

EV contributed to the conceptualization, literature review, synthesis of literature, and draft writing. FH-B, PE, AM, PG, and SF contributed to the conceptualization, article identification, and review of drafts. SF and FH-B additionally contributed to the identification and refinement of recommendations. All authors contributed to the article and approved the submitted version.
